# Differences between adjusted vs. non-adjusted loads in velocity-based training: consequences for strength training control and programming

**DOI:** 10.7717/peerj.10942

**Published:** 2021-03-23

**Authors:** Pedro Jiménez-Reyes, Adrian Castaño-Zambudio, Víctor Cuadrado-Peñafiel, Jorge M. González-Hernández, Fernando Capelo-Ramírez, Luis M. Martínez-Aranda, Juan J. González-Badillo

**Affiliations:** 1 Centre for Sport Studies, Rey Juan Carlos University, Madrid, Spain; 2 Department of Physical Education, Sport and Human Motricity, Universidad Autónoma de Madrid, Madrid, Spain; 3 Faculty of Health Sciences, Universidad Europea de Canarias, La Orotava, Tenerife, Spain; 4 Faculty of Education Sciences, SPORT Research Group (CTS-1024), CERNEP, University of Almeria, Almeria, Spain; 5 Faculty of Sport.Neuromove Research Group, Catholic University of San Antonio, Murcia, Spain; 6 Physical Performance & Athletic Research Center, Faculty of Sports Science, Pablo de Olavide University, Sevilla, Spain

**Keywords:** Velocity-based strength training, Full squat, Performance, Velocity specificity, Resistance training

## Abstract

Strength and conditioning specialists commonly deal with the quantification and selection the setting of protocols regarding resistance training intensities. Although the one repetition maximum (1RM) method has been widely used to prescribe exercise intensity, the velocity-based training (VBT) method may enable a more optimal tool for better monitoring and planning of resistance training (RT) programs. The aim of this study was to compare the effects of two RT programs only differing in the training load prescription strategy (adjusting or not daily via VBT) with loads from 50 to 80% 1RM on 1RM, countermovement (CMJ) and sprint. Twenty-four male students with previous experience in RT were randomly assigned to two groups: adjusted loads (AL) (*n* = 13) and non-adjusted loads (NAL) (*n* = 11) and carried out an 8-week (16 sessions) RT program. The performance assessment pre- and post-training program included estimated 1RM and full load-velocity profile in the squat exercise; countermovement jump (CMJ); and 20-m sprint (T20). Relative intensity (RI) and mean propulsive velocity attained during each training session (V_session_) was monitored. Subjects in the NAL group trained at a significantly faster V_session_ than those in AL (*p* < 0.001) (0.88–0.91 vs. 0.67–0.68 m/s, with a ∼15% RM gap between groups for the last sessions), and did not achieve the maximum programmed intensity (80% RM). Significant differences were detected in sessions 3–4, showing differences between programmed and performed V_session_ and lower RI and velocity loss (VL) for the NAL compared to the AL group (*p* < 0.05). Although both groups improved 1RM, CMJ and T20, NAL experienced greater and significant changes than AL (28.90 vs.12.70%, 16.10 vs. 7.90% and −1.99 vs. −0.95%, respectively). Load adjustment based on movement velocity is a useful way to control for highly individualised responses to training and improve the implementation of RT programs.

## Introduction

Resistance training (RT) is recognized as an effective method for increasing strength, power and muscle hypertrophy. It has also been considered a key component of exercise programs designed for improving health status and overall fitness (Pareja-Blanco et al., 2014). The adaptations achieved through RT depend on the applied stimulus (Spiering et al., 2008). Therefore, it is important to monitor variables such as volume, absolute and relative intensity (RI), number of repetitions, sets and resting time between sets according to FITT principles during RT programs. For this reason, the optimal combination of training variables is especially interesting to strength and conditioning coaches and researchers aiming to maximize dynamic strength gains (Marshall, McEwen & Robbins, 2011).

Intensity generally has been acknowledged as an essential variable in terms of improving strength (Kraemer & Ratamess, 2004). Several authors and strength and conditioning coaches have shown their interest on how to monitor and establish RT intensity in a daily context objectively (Baechle & Earle, 2008). Traditionally, the one-repetition maximum (1RM) approach has been widely used to prescribe and monitor exercise intensity during conventional loading-based RT. In recent years, this method has been one of the most widely recognized tools for prescribing and designing RT programs because of the ease of programming RI in training sessions. However, during the training process, athletes experience daily variations in performance, and the 1RM values for a given subject and exercise may vary between sessions (González-Badillo et al., 2011; Dorrell, Smith & Gee, 2019). Thus, it cannot be guaranteed that the loads (%1RM) being used in each particular training session truly represent the intended ones if the 1RM is not measured at the beginning of each session. Testing 1RM several times to adjust loads during the training period may interfere with the estimated training stimulus due to the significant effort that 1RM represents, resulting in a non-specific stimulus that could interfere negatively in physical performance improvement (González-Badillo & Sánchez-Medina, 2010). For this reason, the difficulty of determining the differences between performed and previously programmed intensities represents a typical concern for practitioners and training decision making processes are very limited due to the existing lack of knowledge of the actual shape and fatigue state of the athlete.

Considering that maximal strength measured by 1-RM can fluctuate daily due to fatigue or increase in short-time due to training adaptation, an outdated 1-RM as a reference to prescribe training loads can represent an important limitation for coaches. Overall, practitioners using traditional %1RM load prescription methods within a periodised model, will aim to account for fluctuations in maximal strength, however given their highly individualised nature it can be near impossible to predict the magnitude of these fluctuations. For example, during a high volume and high frequency training block, coaches may reduce the load used as they understand individuals are likely carrying residual training-induced fatigue session-to-session. Yet precisely the specific fatigue response, and its impact of maximal strength is impossible to quantify without methods such as some authors have proposed as the use of the recently developed velocity-based RT method (VBT). Although recent researches have proposed adjusting training loads considering the method known as “Rating of Perceived Exertion Repetitions-in-Reserve” (RPE-RIR) as an alternative to account the possible individual’s day-to-day fluctuation (Zourdos et al., 2016; Helms et al., 2016; Helms et al., 2017) recent evidence has highlighted some limitations of these methods (Mansfield et al., 2020). To address these issues, some authors have proposed the use of the well-known velocity-based RT method (VBT) which could allow for manipulating training loads within a training session objectively (Weakley et al., 2020a). Therefore, the daily training load can be matched to the previously programmed intensity, since it is acknowledged that each %1RM corresponds to a specific mean concentric velocity. This finding has been reported and used in several studies that achieved similar results, although some studies propose individualizing the load-velocity relationship for each subject (González-Badillo & Sánchez-Medina, 2010; Sánchez-Medina et al., 2017; García-Ramos et al., 2019; García-Ramos & Janicijevic, 2020). In this sense, VBT differs from traditional approaches by modifying training load variables to match the pre-programmed ones prior to a given training session, rather than assigning them based on a 1RM pre-test. Together with 1-RM, a common practice to assess strength gains is the measurement of explosive performance via countermovement jump (CMJ) height and sprint time after a training period (Pareja-Blanco et al., 2017b). Furthermore, neuromuscular fatigue generated during resistance training sessions has been monitored through velocity loss (VL) (Sánchez-Medina & González-Badillo, 2011, Sánchez-Moreno et al., 2017).These parameters may contribute to improving current knowledge of the adaptations produced using VBT protocols. Since resistance training is used by a wide range of populations and its benefits to non-athletes have been well documented (Ratamess et al., 2009)

However, despite the potential usefulness of VBT in strength training, to the best of our knowledge one study by Banyard et al. (2020) has used the VBT method to compare the effects of matching the absolute load to a previously programmed RI with the same training program without adjusting the RI, on training stimulus and adaptations. To date, Banyard et al. (2020) analyzed the effects of a periodized intervention comparing 1RM load percentage-based training (PBT) vs. VBT on maximal strength, CMJ and sprint. Banyard’s study used a similar experimental approach comparing the PBT vs. VBT in highly experienced resistance-trained males with a ratio of 1RM squat/body mass of 1.60. Note that the VBT method may be highly accurate in this population since the 1RM is likely to be quite consistent across the training intervention. However, in non-experienced subjects, this tendency could be different due to an evolution of the 1RM during the training intervention presenting a gap between what has been programmed and actually performed because of the inexperience and potential strength gains due to the learning effects compared to such experienced athletes. In addition, those high performance athletes who combine endurance and resistance training and those whose performance depends directly on the weight lifted as in Weightlifting or Crossfit need a better understanding of the control of their training loads. Thus, to date, no previous studies have investigated the development in physical performance generated from the use of two different stimuli derived from either a previously planned program or the daily adjustments in load according to VBT related decisions in non-experienced subjects. Therefore, The main purpose of this study was to analyze and compare training-induced adaptations in 8-week squat RT programs with different training load prescriptions strategies; one group using 1RM as a reference to prescribe each training session load and the other using VBT to adjust each training session load and monitor the evolution of RI and the attained mean velocity during each training session in unexperienced males. Thus, one group would use the initial 1RM as a reference to prescribe loads for every training session and the VBT group would adjust individual training loads in each training session. The secondary purpose was to compare the effects of both training programs on maximal strength, load velocity profile, sprint performance and CMJ height. We hypothesized that intensity (in both groups) would differ between groups in each training session over the 8-weeks because the group without session-adjusted individuals training loads would train with an outdated 1-RM reference in few sessions. In addition, that would negatively affect the neuromuscular adaptations achieved during the program likely due to the use of either lower or greater RI than optimal and, in the latter case, an increase in accumulated fatigue throughout the intervention program.

## Methods

### Participants

Twenty-four physically active male sport sciences students aged 23.1 ± 4.1 years, body mass 76.3 ± 6.4 kg, height 1.81 ± 0.06 m and SQ 1RM load 80.3 ± 15.2 kgs voluntarily participated in the study. All participants had at least 2 years of RT experience with a minimum of 3 sessions per week. During the duration of this research, the participants were only doing the training prescribed by us. Participants had to prove they could perform a strict and correct technique to be accepted in this investigation. No physical limitations, health problems or musculoskeletal injuries that could affect testing or training were found after a medical examination. No participants declared the use of drugs, medications or dietary supplements known to influence physical performance. Participants were randomly assigned to one of the two designated groups, which were counterbalanced using the subjects’ initial 1-RM in pre-test and allocated to either the AL (*n* = 13) or NAL (*n* = 11) group. Initially, both groups had the same number of participants (13), but two participants had to abandon the study during the training period; one got a foot injury, and one dropped out due to incompatibility with training times. One group performed programmed VBT with relative loads, ranging from 50 to 80%, adjusting the relative loads in each session according to velocity (AL), while the other group did not adjust relative loads and worked using absolute programmed loads (NAL). Once informed about the purpose, testing procedures and research potential risks, all subjects gave their written informed consent to participate in this study, which was approved by the local ethical committee and conducted in agreement with the Declaration of Helsinki. The Catholic University of San Antonio of Murcia UCAM Ethics Committee granted ethical approval to carry out the study within its facilities. No physical limitations or musculoskeletal injuries that could affect the study were reported.

### Study design

The present study used a longitudinal pre-post design with testing sessions separated by 8 weeks. All tests were conducted at the same time, from 17:00 to 21:00 to the pre and pos *t*-test week. Subjects trained twice a week for 8 weeks, completing a total of 16 sessions with a minimum 48-hour rest period between sessions. One week after the final training session, the pos *t*-test was performed. A progressive RT program that comprised only the SQ exercise was used (Table 1). RI programmed ranged between 50% (session 1) to 80% (session 16). NAL group lifted the corresponding absolute load to match with the programmed RI based on their initial 1-RM with velocity monitored, but not used for prescription. However, the AL group lifted with loads adjusted to the programmed RI in each session to reach the planned sessional target. For example, in session one, NAL group trained with the absolute load that corresponded to 50% 1-RM load, whereas AL group used the load necessary to achieve the sessional target velocity of 1.13 m/s which corresponded to 50% 1-RM as determined at baseline. The RT program was designed with the same relative loading magnitude (%1RM) expressed with a target velocity to attain in each training session. Both groups trained with the same matched sets and repetitions. The AL group matched the programmed RI to the target velocity by modifying the absolute loads in each training session (Table 1). The NAL group performed the programmed training without adjusting the absolute load to the programmed target velocity. Their training only took into account the initial 1RM as a reference and used absolute loads that represented the pre-programmed relative intensities (based on the initial 1RM test) for each session. Velocity was monitored for both groups for all repetitions during all the training sessions. Sessions were performed in a research laboratory under the direct supervision of the investigators, at the same time of day (±1 h) for each subject and under controlled environmental conditions (20 °C and 60% humidity). Subjects were required not to engage in any other type of strenuous physical activity or exercise training while they were involved in the present investigation. Every session was supervised by the researchers (specialist in strength and conditioning) in order to certify proper technique was used and to help with the selection of loads.

**Table 1:** Characteristics of the strength training performed in squat by AL and NAL group.

Scheduled	Wk 1	Wk 2	Wk 3	Wk 4	Wk 5	Wk 6	Wk 7	Wk 8
**Sets ×reps**								
*Session 1*	3 × 6	3 × 5	4 × 4	3 × 4	4 × 3	4 × 4	4 × 2	3 × 4
*Session 2*	3 × 8	3 × 6	4 × 5	3 × 5	3 × 4	3 × 5	4 × 3	3 × 2
*Target MVP (m s*^−1^*)*	1.13	0.98	0.90	0.82	0.75	0,75	0.68	0.68
*(% 1RM)*	(∼50% RM)	(∼60% RM)	(∼65% RM)	(∼70% RM)	(∼75% RM)	(∼75% RM)	(∼80% RM)	(∼80% RM)
***Actually Performed***								
*AL Session 1*	1.13 ± 0.02	0.96 ± 0.02	0.89 ± 0.04	0.82 ± 0.02	0.75 ± 0.02	0.74 ± 0.01	0.68 ± 0.02	0.67 ± 0.01
	(∼50% RM)	(∼61% RM)^&&^	(∼66% RM)^&&&^	(∼70% RM)^&&^	(∼75% RM)^&&&^	(∼75% RM)^&&&^	(∼80% RM)^&&&^	(∼80% RM)^&&&^
*AL Session 2*	1.11 ± 0.03	0.95 ± 0.02	0.87 ± 0.02	0.83 ± 0.03	0.73 ± 0.02	0.73 ± 0.01	0.67 ± 0.02	0.67 ± 0.01
*NAL Session 1*	1.11 ± 0.02	1.00 ± 0.04	0.96 ± 0.07	0.91 ± 0.09	0.90 ± 0.12	0.90 ± 0.10	0.88 ± 0.12	0.90 ± 0.11
	(∼51% RM)	(∼59% RM)	(∼61% RM)^*^	(∼64% RM)^***^	(∼65% RM)^***^	(∼65% RM)^***^	(∼66% RM)^***^	(∼64% RM)^***^
*NAL Session 2*	1.12 ± 0.03	1.00 ± 0.05	0.97 ± 0.08	0.93 ± 0.11	0.90 ± 0.12	0.91 ± 0.11	0.88 ± 0.11	0.91 ± 0.11
	(∼50% RM)	(∼58% RM)	(∼60% RM)^**^	(∼63% RM)^***^	(∼65% RM)^***^	(∼64% RM)^***^	(∼66% RM)^***^	(∼64% RM)^***^
***Velocity Loss***								
*AL Session 1*	10.05 ± 2.70	11.15 ± 2.93	11.04 ± 3.09	11.18 ± 3.24^†^	9.01 ± 3.47^†^	13.92 ± 6.30^††^	6.74 ± 2.40^††^	11.54 ± 3.10^†††^
*AL Session 2*	13.34 ± 2.83	12.23 ± 4.01	11.48 ± 2.64^†^	12.53 ± 3.06^†^	10.46 ± 5.75^†^	14.72 ± 4.67^††^	11.36 ± 3.91^††^	5.85 ± 2.93^†^
*NAL Session 1*	10.35 ± 2.32	10.59 ± 2.94	8.78 ± 4.00	8.20 ± 2.81	6.43 ± 1.65	7.65 ± 2.63	3.19 ± 1.46	4.25 ± 1.88
*NAL Session 2*	13.09 ± 3.48	11.84 ± 3.99	8.49 ± 2.79	8.94 ± 2.54	6.41 ± 2.08	8.34 ± 3.55	5.22 ± 1.82	2.68 ± 1.67

**Notes.**

Values are shown as mean ± SD.

Abbreviations AL Adjusted Load NAL Not Adjusted Load Wk week MVP Mean Propulsive Velocity in the concentric phase during squat RM Repetition Maximum m s^−1^ meter ×second; % percentage

Intra-group significant differences for NAL group from difference between target velocity and actually performed.

* *p* < 0.05.

** *p* < 0.01.

*** *p* < 0.001.

Inter-group significant differences between AL and NAL for Velocity Loss each training session.

† *p* < 0.05.

†† *p* < 0.01.

††† *p* < 0.001 and *V*_session_.

& *p* < 0.05.

&& *p* < 0.01.

&&& *p* < 0.001.

### Equipment and data acquisition

The SQ test was performed in a Smith machine (Multipower Fitness Line, Peroga, Spain) that allowed a smooth vertical displacement of the bar along a fixed vertical path. The velocity values were measured with a linear velocity transducer (T-Force System, Ergotech, Murcia, Spain) providing immediate feedback and saving the data in the program for later analysis. To measure the jump height was used an infrared timing system (Optojump, Microgate, Bolzano, Italy) and sprint times were measured using photocells (Polifemo Radio Light, Microgate, Bolzano, Italy).

### Testing procedures

#### Load-velocity squat profile

Full-squat 1RM strength was determined using an incremental loading test. Warm-up consisted of 5 min of treadmill running at 10 km/h, 5 min of lower body joint mobilization exercises, and two sets of eight and six SQ repetitions (3-min rests) with loads of 20 and 30 kg, respectively. Exactly the same warm-up and progression of absolute loads for each subject was used at Pre and Post-tests. The SQ was performed as described in recent studies (Pareja-Blanco et al., 2014; Pareja-Blanco et al., 2017a; Pareja-Blanco et al., 2017b); (González-Badillo et al., 2016). Auditory feedback based on eccentric distance travelled was provided to help each subject in both groups to reach his previously determined squat depth. While the eccentric phase was performed at a controlled mean velocity (Vmean) (∼0.50–0.65 m/s), subjects were required to execute the concentric phase at maximal intended velocity. Initial load was set at 20 kg and was progressively increased in 10 kg increments until the attained mean propulsive velocity (MPV) was <0.8 m s^−1^. Thereafter, the load was individually adjusted with smaller increments (5 down to 2.5 kg) so that 1RM could be precisely determined. Three repetitions were executed for light (≤ 50% 1RM), two for medium (50–80% 1RM) and only one for heavy loads (>80% 1RM) (Sanchez-Medina, Perez & Gonzalez-Badillo, 2010; Pareja-Blanco et al., 2017b). Strong verbal encouragement was provided to motivate participants to give a maximal effort. Inter-set recoveries ranged from 3 min (light) to 5 min (heavy loads). As suggested by recent studies (Pareja-Blanco et al., 2014; Pareja-Blanco et al., 2017b; González-Badillo et al., 2016), only the best repetition at each load, according to the criterion of fastest MPV, was considered for subsequent analysis. All velocity measures reported in this study corresponded to the Vmean of the propulsive phase of each repetition. The propulsive phase was defined as the fraction of the concentric phase where the barbell acceleration was greater than the acceleration due to gravity (Sanchez-Medina, Perez & Gonzalez-Badillo, 2010). In addition to 1RM strength, three other variables obtained from this progressive loading test were used to analyse the extent to which the two training interventions (AL vs NAL) affected different parts of the load-velocity relationship: Average MPV of (a) all absolute loads, as well as loads which resulted in an MPV (b) above or equal too, and (c) below 1 m s^−1^.

#### Sprinting and jumping tests

Jump height and sprint time can be used as indicators of explosive performance, because improved explosive performance is related to an increase in the jump height and better sprint performance times (Pareja-Blanco et al., 2017b). After a standardized warm-up, consisting of 10 min of jogging, dynamic stretching and preparatory vertical jumps, participants performed five maximal CMJs. During the CMJ, the subject was instructed to rest his hands on his hips while performing a downward movement to 90° of knee flexion followed by a vertical jump with maximum effort. Since the depth and the velocity of the descent affects performance in the CMJ (Pérez-Castilla et al., 2019b), we proposed that the subjects should arrive at the same angle of knee flexion prior to the jump, which in addition, means an individualized depth. All subjects were instructed to keep their knees straight during the flight phase of the jump and to land in an upright position. Attempts were separated by 20 s of passive rest. The highest and lowest CMJ height values were discarded, and the resulting average was kept for analysis (Claudino et al., 2017). After the jump test, two maximal 20m sprints separated by a 3 min rest were performed on an indoor running track. Photocell timing gates were placed at 0 and 20 m to determine the time to cover 0–20 m (T20). A standing start with the lead-off foot placed 1 m behind the first timing gate was used. Subjects were required to give an all-out maximal effort in each sprint and the best of both trials was kept for analysis. The same warm-up protocol incorporating several sets of progressively faster 20 m running accelerations was followed at Pre and Post.

#### Resistance training program

Descriptive characteristics of the RT program are shown in Table 1. Both AL and NAL groups trained using only the SQ exercise as previously described. The programmed RT was the same in terms, number of sets (three), and inter-set recoveries (4 min) for both groups in each training session. All repetitions were objectively quantified and monitored by the velocity achieved in each repetition for both groups during the whole RT program for all training sessions. In order to individualize the training load, MPV was used during the warm-up exercises to adjust the load via the load-velocity relationship proposed by Sanchez Medina et al. 2011. Thus, a target MPV to be attained in the first (usually the fastest) repetition of the set in each session was used as an estimation of % 1RM, as follows: 1.13 m s^−1^ (∼50% 1RM), 0.98 m s^−1^ (∼60% 1RM), 0.90 m s^−1^ (∼65% 1RM), 0.82 m s^−1^ (∼70% 1RM), 0.75 m s^−1^ (∼75% 1RM), and 0.68 m s^−1^ (∼80% 1RM). This MPV attained during each training session was summarised as (V_session_). The difference between AL and NAL groups was that for the AL group, the absolute load (kg) was individually adjusted to match the velocity associated (±0.03 m s^−1^) with the %1RM intended for each session. For the NAL group, the absolute load for each session was established beforehand by taking the initial 1RM in the Pre-test as a reference. Thus, both groups performed the same number of repetitions per set and completed the same total number of repetitions during the training program but differed in whether or not the training loads were adjusted during training. Thus, for both groups, velocity was monitored in every training session. In NAL this was done only to analyse the mismatch between what was programmed and what was actually performed during RT. For AL, velocity was checked to match the programmed RI with that performed daily, ensuring there was no mismatch in this group. This mismatch is what we considered to be the “gap” between pre-programmed RI and RI actually performed. Moreover, in each session, the VL (difference between the fastest and slowest repetition in each set) was calculated for each group as a measure of neuromuscular fatigue (Sánchez-Medina & González-Badillo, 2011).

Finally, during training, subjects received immediate velocity feedback from the measurement system while being encouraged to perform each repetition at maximal intended velocity (Weakley et al., 2018).

### Statistical analyses

All data are shown as mean ± standard deviation (SD). Normality was checked with the Shapiro–Wilk test before analyses. Data were analysed using a 2 ×  2 factorial ANOVA with Bonferroni’s post-hoc comparisons using one inter factor (AL vs NAL) and one intra factor (Pre vs Post). Statistical significance was established at the *P* ≤ 0.05 level. Effect sizes (ES) were calculated using Hedge’s g on the pooled SD. All statistical analyses were performed using SPSS software version 18.0 (SPSS Inc., Chicago, Illinois, USA).

## Results

**Figure 1:**
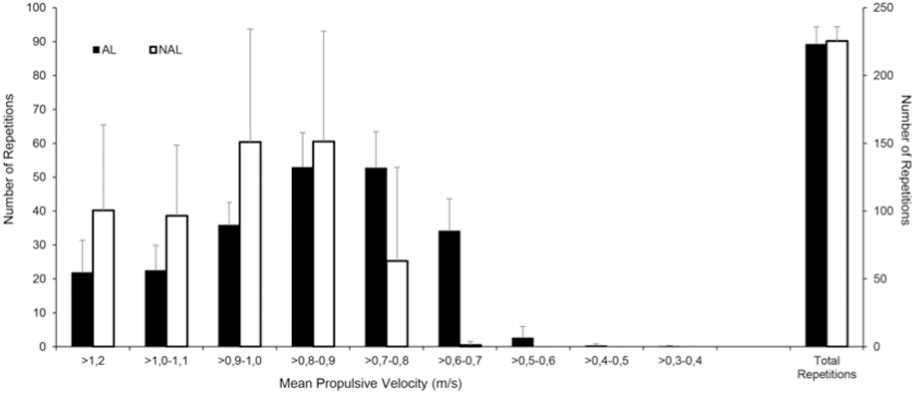
Number of repetitions in the squat exercise. Number of repetitions in the squat exercise performed in each velocity range, and total number of repetitions completed by both training groups.

**Figure 2:**
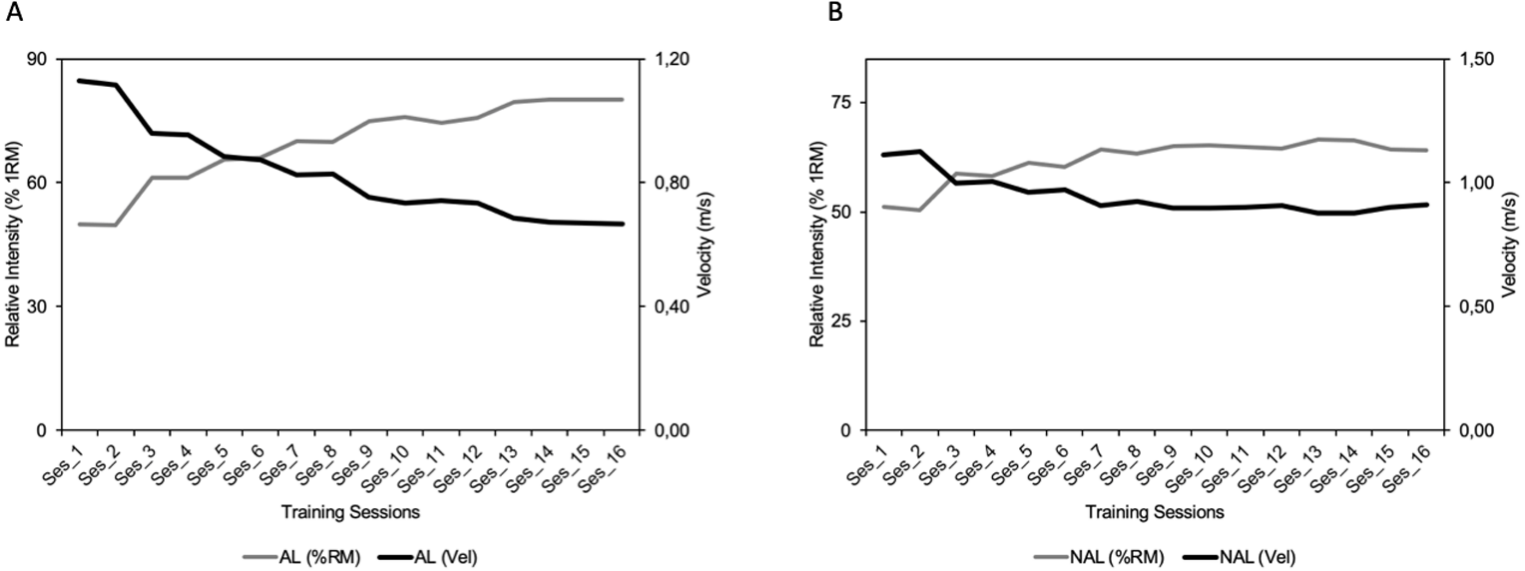
Evolution of relative intensity and mean velocity. Evolution of relative intensity and mean velocity attained during each training session by Adjusted Load (A) and Non-Adjusted Load (B) training groups.

**Figure 3:**
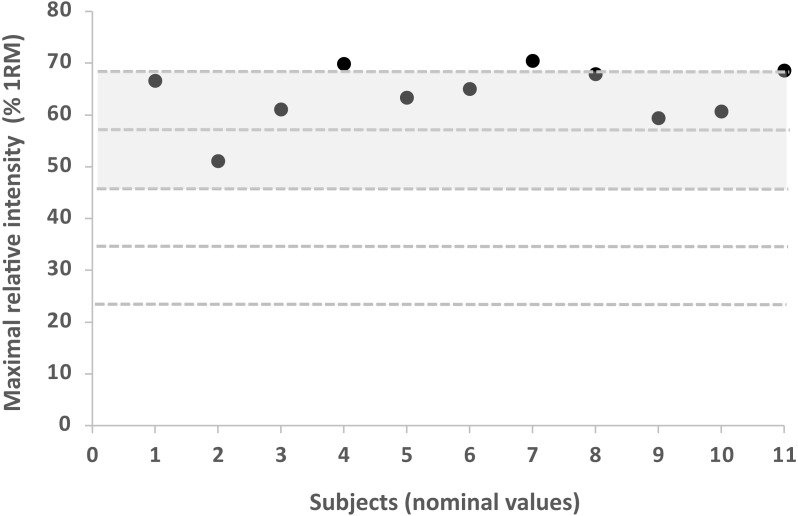
Maximal relative load achieved in the last training session. Maximal relative load achieved in the last training session for each subject in the Non-Adjusted Load group. *X*-axis values represent nominal values. The maximum intensity programmed was 80% RM.

No significant differences were found between the two groups in Pre for any of the variables analysed. Table 1 shows the descriptive characteristics of the training performed by both groups, the evolution of the RI, V_session_ performed and VL during the training program. The repetitions performed in different velocity ranges for each group are shown in Fig. 1. Subjects in the NAL group trained at a significantly faster average MPV in session (V_session_) than those in AL (0.86 ± 0.11 vs 0.82 ± 0.13 for NAL and AL, respectively; *p* < 0.001). These differences are especially evident when examining the difference between programmed and performed RI and V_session_ (i.e., 80% vs. 64% RM and 0.91 vs. 0.67 m/s, representing a difference of 20% and 35.8% respectively for session 16) (Table 1 & Fig. 2). In this case, the NAL group did not achieve the maximum intensity programmed (80% RM) for the last training sessions, performing ∼64% RM maximum (Fig. 3). When analysing the existing gap between the programmed V_session_ for each session and the performed one, a short and stable range (−3.98 to 1.84%) was found for the AL group throughout the whole intervention. However, this gap between the intended and performed training velocity was increasingly higher for the NAL group, starting the statistically significant differences with a 6.9% (ES: 1.02) gap for session 5 and concluding the intervention with a difference of 32% (ES: 2.21) for session 16. (Fig. 3). V_session_ as reflected by the NAL group matched the expectations during the first sessions (−1.74, −0.71, 2.70 and 3.02% RI gap for sessions 1 to 4 respectively; ES: 0.52, 0.52, 0.68, 0.71, respectively), but from the fifth session onwards (6.9%), a turning point could be observed between the programmed and reflected RI, with an accentuated trend that was not redirected.

Significant differences were detected from session 6 onwards for VL (*p* < 0.05 to *p* < 0.001) between groups, with the NAL group showing significantly lower losses in terms of VL for sessions 6 to 16.

We analyzed the effects on the load-velocity relationship and how adjustment or lack of adjustment of the loads affected the load-velocity curve by measuring the average MPV attained with lighter (AV >1m/s), heavier (AV <1m/s), and all absolute loads (AV) and found significant differences between AL and NAL groups for the entire load-velocity curve and those loads moved at velocities greater than 1m/s (corresponding to light loads) (Table 2).

**Table 2:** Changes in the selected neuromuscular performance variables from pre- to post- training for each group.

	**AL**				**NAL**			
****	**Pre**	**Post**	Δ**(%)**	**ES**	**Pre**	**Post**	Δ**(%)**	**ES**
**AV(m s**^−1^)	0.98 ± 0.11	1.17 ± 0.10^***^	19.58 ± 0.20	1.80	0.89 ± 0.10	1.20 ± 0.10^***^	35.12 ± 0.31^†^	3.10
**AV ≥1 (m s**^−1^)	1.34 ± 0.08	1.43 ± 0.08^***^	6.34 ± 0.06	1.12	1.28 ± 0.10	1.36 ± 0.10^***^	7.27 ± 0.09^†^	0.80
**AV<1 (m s**^−1^)	0.69 ± 0.08	0.81 ± 0.08^***^	18.53 ± 0.19	1.50	0.72 ± 0.10	0.85 ± 0.10^***^	10.37 ± 0.06	1.30
**1RM (kg)**	85.52 ± 16.47	96.39 ± 16.18^***^	12.70 ± 0.06	0.67	74.07 ± 11.35	95.47 ± 16.93^***^	28.90 ± 0.12^†^	1.48
**CMJ (cm)**	37.35 ± 3.47	40.31 ± 3.71^***^	7.90 ± 0.06	0.82	32.86 ± 5.36	38.16 ± 5.05^***^	16.10 ± 0.07^†^	1.02
**T_10 (s)**	1.86 ± 0.06	1.84 ± 0.06^**^	−1.20 ± 0.0	0.50	1.89 ± 0.08	1.85 ± 0.08^**^	−2.26 ± 0.0	0.53
**T_10-20 (s)**	1.31 ± 0.02	1.30 ± 0.02^**^	−0.59 ± 0.0	0.50	1.31 ± 0.02	1.29 ± 0.04^***^	−1.60 ± 0.0	0.63
**T_20 (s)**	3.17 ± 0.08	3.14 ± 0.08^***^	−0.95 ± 0.01	0.37	3.20 ± 0.10	3.14 ± 0.10^***^	−1.99 ± 0.01^†^	0.60

**Notes.**

Data are shown as mean ± SD. Summary of the effects of different trainings protocols on force-related parameters. Intra-group significant differences from pre to post training.

* *P* < 0.05.

** *P* < 0.01.

*** *P* < 0.001.

Inter-group significant differences between Adjusted Load and Not Adjusted Load.

† *P* < 0.05.

Abbreviations AL Adjusted Load NAL Not Adjusted Load AV average “Mean Propulsive Velocity” (MPV) attained against absolute loads common to pre- and post-test in the squat progressive loading test AV ≥ 1 average MPV attained against absolute loads common to pre- and post-test that were moved equal or faster than 1 m/s AV < 1 average MPV attained against absolute loads common to pre- and post-test that were moved slower than 1 m/s 1RM Repetition Maximum in squat m s ^−1^ meter × second CMJ countermovement jump height T_10_ 10-m sprint time T_10−20_ 10–20 m lapse sprint time T_20_ 20-m sprint time

In addition, the gaps between intended and performed training velocity not only contributed to a clear difference in RI but were accompanied by differences in neuromuscular performance as shown in the expression of force production during SQ, CMJ and sprint in each group. Significant improvements (*p* < 0.001) were observed for SQ, CMJ and sprint performance after training for both groups (Table 2). Despite this clear improvement for each group, the results seemed to be more positive for the NAL group, with significant differences between AL and NAL groups for SQ, CMJ and sprint performance.

## Discussion

The purpose of this study was to compare two resistance training protocols differentiated only by adjusting or not adjusting RI to a pre-established reference (1RM) during 8 weeks of RT. The aim was to determine whether the two protocols would produce different strength and performance outcomes. Our first hypothesis; that greater strength gains would be achieved by the %1RM-based group (NAL), was supported. The main finding of this study was that the NAL group, which performed the same volume as the AL group but with lesser VL induced in each training session, showed greater strength gains in the full-squat exercise, CMJ, and sprint than the AL group (Table 2). In fact, NAL experienced a VL of 7.78% whereas AL group suffered a VL of 11.04% on average for the entire training program. It is important to note that both groups performed the same number of repetitions in each training session. These findings may indicate that a stimulus characterized by a low degree of induced fatigue (NAL) and high velocity of the repetitions within the set may be enough to induce strength adaptations in a non-experienced population. Several studies have reported the effects of VL on strength gains (Pareja-Blanco et al., 2017b; González-Hernádez et al., 2020), highlighting the usefulness of VBT in RT. These studies have been performed considering 1RM as a reference to prescribe the RI for each training session, adjusting absolute loads to the corresponding pre-programmed RI. Banyard et al. (2020) compared a 6-weeks training period of RT using either PBT or VBT adjusting loads daily to perform identical relative intensities using individual load-velocity relationships for each subject in the VBT group. The increased differences between PBT and VBT identified in Banyard’s study may be attributed to the decreased training status of participants. Indeed, the increased training status of the participants examined by Banyard et al. likely means 1RM is more stable than for the participants of the current study, hence reducing the gap between programmed and performed RI. The protocols of the current study, however, differ from Banyard et al. (2020), who used a fixed-dose for all the training intervention of 5 sets of 5 repetitions for each training session during 6-weeks (3 sessions/week) with a total volume of 25 reps per session, 75 reps per week and a total volume of 450 repetitions for the entire training intervention. Instead in the present study, we used a progression of loads which was adapted during 8-weeks (2 sessions/week) to adapt according to the progressive increase of RI and sets and reps was not always the same as in Banyard’s study. In summary, in our study the volume was ranged from 18 reps (week 7) to 42 reps (week 1) what was a total volume of 231 repetitions for the entire training intervention. Considering that loads progressed over time, it is important to note from a practical standpoint that our results may be more ecologically valid and better represent the trends that would be observed in a real-world setting. Thus, our results could be complementary to those reported by Banyard et al. (2020) and a really practical information for many coaches when aiming to optimize training time in some team-sports contexts where remains challenging due to congested competition calendars. Moreover, Banyard et al. (2020) monitored each training session with RPE scores, which although is interesting and very practical, it is limited as it does not exactly measure the effect of the actual fatigue produced by each training session on performance in each group. In the study mentioned above, each training session was monitored by velocity loss what has been reported to be a reliable and good indicator and marker of fatigue induced by a strength training load (González-Badillo et al., 2017; Pareja-Blanco et al., 2017b; Mora-Custodio et al., 2018; Rodríguez-Rosell et al., 2020b). Thus, our results highlight the key role of monitoring RT through VBT in order to get a better understanding of fluctuations produced by the two RT strategies in non-experienced subjects. Our results suggest the importance of assessing the daily load adjustment for a pre-programmed RI and the level of matching or mismatching between what is pre-programmed and what is actually performed (gap). Thus, it is worth noting that rather than the confirmed usefulness of adjusting individual training loads through a VBT method. The novelty of this study is that when comparing both strategies it is possible to precisely assess the specific RI which is actually performed and determine the exact moment throughout the training period in which there exists a difference (gap) between the programmed training and that actually performed in non-experienced subjects. In the NAL group we could assess the gap between pre-programmed RI and RI actually performed. In the NAL group this gap increased as the training sessions progressed, becoming significant from session 5 to the last session. (Table 1; Fig. 3).

Velocity-based training has been recognized as a very reliable and practical method for determining RI (and in turn the level of effort created by a given load) (González-Badillo et al., 2017; Weakley et al., 2020b). The practical point of this method is that it can ensure that the relative loads (%1RM) being used in any given training session truly represent the intended ones (González-Badillo & Sánchez-Medina, 2010). With this novel approach, the training load for each session is set to match a given %1RM, which has a corresponding mean concentric velocity. The vast majority of research studies in the sport sciences field (Griffiths et al., 2019; Pritchard et al., 2019) have prescribed training intensities according to 1RM values from which each relative load was obtained prior to exercise. This approach is understandable since authors such as (Seo et al., 2012) established the reliability of maximum strength capabilities after evaluation using the RM test. However, it should be noted that these results were obtained by maintaining the subjects in a stable assessment environment, which can rarely be found once the dynamics of training and competition come into play (Bishop, Jones & Woods, 2008; Doeven et al., 2018; Hughes et al., 2019). Hence, it is essential to understand that human neuromuscular performance is exposed to an endless number of external variables (e.g., mood, sleep, motivation or stress) capable of modifying the potential gains of performance both acutely and chronically (Dalgas et al., 2010). Thus, training with pre-set/pre-established loads may involve different levels of effort than desired, given the daily modification of the 1RM (Banyard et al., 2020).

Despite the great interest in new resistance training approaches such as VBT (Courel-Ibáñez et al., 2019; Pareja-Blanco et al., 2019; Pérez-Castilla et al., 2019a; Weakley et al., 2020a), there is limited scientific data measuring the real effort applied in each training session (by monitoring movement velocity). This study presented the novelty of assessing VL of a daily adjustment of training loads based on velocity, to match the relative programmed intensity (for the AL group) or using previously determined absolute loads corresponding to the programmed RI without modifying loads daily (for the NAL group) in two RT programs identical with regard to volume (Fig. 1). Generally, studies using percentage-based approaches cannot provide information about the real intensity performed in each training session since athletes are using a pre-programmed RI based on 1RM as the reference for prescribing training. Using the VBT approach means that this problem can be solved since coaches know the real relative intensity performed. Identifying the gap between prescribed and performed RI might indicate whether some individuals could benefit from adjusting or not adjusting the RI in each session as a loading strategy. Considering our results, at the group level the NAL group trained at a higher average mean velocity than AL and showed a progressive gap between the RI performed and the pre-programmed value (Table 2; Fig. 2). Specifically, NAL diverged from AL from session 4 onwards for programmed RI. This resulted in smaller velocity losses and higher mean velocities in each training session (from session 6) that remained higher throughout the rest of the study. Practically, it must be noted that without the use of VBT, the gap between programmed and performed RI observed from the fourth session onwards would have gone undetected. It is worth to note that when this mismatch occurs, different level of fatigue and potential adaptations are experienced to those planned. Thus, this information will be relevant for coaches and practitioners to check the existing individual mismatch between subjects and adapt training accordingly, always knowing the real stimuli induced and performed. The findings of our study suggest that in a moderately trained population at the beginning of a structured strength and conditioning programme, it seems that quality goes over quantity. Interestingly, significant gains in strength and increases in CMJ and sprint performance occurred in both groups, although the majority of the training in NAL occurred with higher velocity and reduced fatigue compared to AL. This provides further evidence that training with less VL may be enough to make significant gains in strength and performance outcomes (Pareja-Blanco et al., 2017b; Galiano et al., 2020), which may allow for a fast recovery when training with moderate to heavy loads (Ogasawara et al., 2013). Thus, our research shows how a daily adjustment of the training load could significantly impact previously designed training programmes (the gap between what is planned and what is actually performed).

The gap in training between the initially tested 1RM (not adjusted) and adjusting to the load based on a daily 1RM showed a significant difference in V_session_, being lower for the unadjusted load group (from session 4) (Table 1; Fig. 3). These results illustrated that not adjusting the load on a daily basis resulted in an increase in the effectiveness of the training, coupled with the use of a load lower than that scheduled for the training session. While both groups completed the volume of training initially planned, RI progressively decreased over time for the NAL group when compared to the AL group, resulting in lesser fatigue (represented by a lower VL in each training session) as the load represented a reduced percentage of the pre-established 1RM (Sánchez-Medina & González-Badillo, 2011) (Fig. 2). This fact has not only been linked to a decrease in neuromuscular and metabolic fatigue but also to a faster rate of neuromuscular recovery and a more suitable hormonal environment (Pareja-Blanco et al., 2018). Although on this occasion, a lower RI brought greater benefits, coaches should be aware that if the adequacy and adaptation of the loads is not correct, the results are likely to be unexpected. This fact can alter considerably the achievement of established objectives in short-, medium-, and even long-term programming, mainly due to the training being carried out in a different way from the programmed approach.

Our results suggest that the implementation or lack of implementation of daily training load adjustment resulted in two training programs that, although otherwise identical, differed in the stimuli provided from the early stages. While most training programs are based on the principles of overload, periodization and load progression, the analysis of these results highlights the major role of a daily assessment and adjustment of the training load, since RM values can be influenced both (i) by the state of readiness -mainly defined by accumulated fatigue and the quality of recovery (short term); and (ii) by adaptations produced as a consequence of training (medium and long term). Thus, the results of this study reinforce the necessity not only of monitoring athletes’ training sessions, but also of adjusting training loads in order to achieve the established plan. This could help to control underlying mismatches in the training process, and increase awareness of the factors that induce neuromuscular adaptations.

It is worth noting that the RI actually performed by NAL was not programmed beforehand since it was the consequence of changing the absolute load in each session, based on an initial 1-RM measurement. Thus, it seems reasonable to program the increase in absolute load according to individual responses through a VBT method. Lastly, from a practical standpoint the increase in absolute load should be a sufficient stimulus to improve performance at the same time as RI remains similar or even decreases. However, if the RI increases at the same pace as the absolute load, performance does not improve and monitoring of movement velocity would be needed in order to improve performance and experience positive training adaptations.

We acknowledge that some limitations exist within this study. Note that a Smith machine has been employed for developing this study and it has been reported differences in load-velocity profiles compared to free-weight exercise (Hughes, Peiffer & Scott, 2020) and it would be considered. If it is well known that a free weight back squat is commonly used in trained population, however novice subjects may benefit from using a Smith machine since it restricts the movement in a strictly vertical motion. Another limitation of this study is that the strength improvement may have been greater in NAL because the actual intensities of 1RM performed were lower than those performed by AL, who worked with adapted relative loads in each training session rather than loads programmed in advance. Interestingly, it could be reasonable to think that this greater improvement in NAL could be due to the use of lighter loads than AL, as Torres-Torrelo et al. reported (Torres-Torrelo, Rodríguez-Rosell & González-Badillo, 2017), but the most likely reason would be that NAL experimented a lower degree of fatigue and also were able to displace the loads at faster velocities (González-Badillo et al., 2017;  Pareja-Blanco et al., 2017c; Mora-Custodio et al., 2018; Rodríguez-Rosell et al., 2020a; Rodríguez-Rosell et al., 2020b) at each training session. However, the adaptations in NAL would differ due to the lesser level of fatigue induced in each training session, as supported by several studies (Pareja-Blanco et al., 2017b; Pareja-Blanco et al., 2017a). However, if this is a limitation of the study, it might also be a limitation of percentage 1RM-based loading in general, as the actual RI performed differed from the intended RI and adaptation through training would be substantially different between individuals. Thus, if we do not check and monitor movement velocity in order to determine the RI during RT it will be hard to control and understand mismatches in the training process, and to understand the mechanisms inducing neuromuscular adaptations.

## Conclussion

This intervention was not intended to test whether one method was better than another. Although NAL provided a slight strength gains in, this does not mean that either adjusting or not adjusting training loads should be considered mutually exclusive for load prescription. Combining these two strategies in strength training could be determinant since it allows strength gains and optimizes adaptation regarding the aims of specific populations, time of the season, the needs for performance, etc. These two strategies in isolation could neither ensure to train with fatigue nor continue with a training based on an outdated 1-RM reference to allow the optimization of training stimuli and to maximize adaptation. Using a NAL strategy would allow for training with each absolute load until this load does not represent the RI programmed. From that time onwards, using an AL strategy in which the new absolute load representing the planned RI may be optimal to keep on the desired evolution. Thus, using AL or NAL strategies could be complementary, and it worth to note that this combination could be effective since it is based on VBT. Thus, this study is expected to contribute to the resistance training field by providing a novel insight on the influence of adjusting or not adjusting the load during a training program. Monitoring RT through VBT in order to get a better understanding of fluctuations produced by RT should be considered by coaches and practitioners, and could result in objective information about the mismatch between what is pre-programmed and what is actually performed. This could provide greater knowledge and control of the underlying adaptive reality for each athlete during the training period, with such applications particularly important for high-performance athletes competing in strength sports or sports involving large amounts of concurrent training. This could provide greater knowledge and control of the underlying adaptive reality for each athlete during the training period. Specially for high performance athletes such as those competing in strength sports or concurrently trained team sport athletes. For this reason, the use of VBT approaches should be considered as a useful tool in optimizing the decision-making process during the planning and implementation of training programs. Lastly, and considering all together, future research should take these adaptations into account when designing training programs since yet a number of unanswered questions with respect to the VBT research should be considered.

##  Supplemental Information

10.7717/peerj.10942/supp-1 Supplemental Information 1 Dataset of all subjects for each variables pre- post training period Contains the pre- and post-values of each variable studied in this research.

## Additional Information and Declarations

### Data Availability

The following information was supplied regarding data availability: Raw data are available as a Supplementary File.
